# Analysis of maternal and perinatal determinants of allergic sensitization in childhood

**DOI:** 10.1186/s13223-020-00467-5

**Published:** 2020-07-31

**Authors:** Samuel Schäfer, Anthony Liu, Dianne Campbell, Ralph Nanan

**Affiliations:** 1 Department of Clinical and Experimental Medicine, Linköping University, Linköping, Sweden; 2 Discipline of Paediatrics and Child Health, Charles Perkins Centre-Nepean, Sydney Medical School-Nepean, The University of Sydney, Penrith, NSW Australia; 3 Immunology and Allergy, The Children’s Hospital at Westmead, Westmead, NSW Australia; 4 Discipline of Child and Adolescent Health, The University of Sydney, Sydney, NSW Australia

**Keywords:** Perinatology, Atopy, Assisted conception, Skin prick test, Fetal development

## Abstract

**Background:**

Non-communicable diseases, such as allergies, are influenced by both genetic and epigenetic factors. Perinatal determinants conceivably modify the epigenetic makeup of the developing fetal immune system preventing or predisposing the development of allergies. The aim of this study therefore was to identify independent perinatal factors associated with allergic sensitization in childhood.

**Methods:**

In a single center retrospective case-cohort study electronic obstetric medical records and available skin prick testing results of children were analyzed. For the analysis 286 skin prick test positive (sensitized) children [median (IQR): 3.47 (1.70–7.34) years] were compared with data from all remaining live births in the obstetric cohort (n = 66,583).

**Results:**

Sensitized children more frequently had a mother born in Asia (19.1% vs. 10.2%; P < 10–6). Applying backward elimination logistic regression, seven out of 23 initially entered perinatal factors remained in the model. High maternal age (> 35 years; OR: 1.912; P < 0.001), male offspring sex (OR: 1.423; P < 0.01) and assisted conception (OR: 1.771; P < 0.05) increased the risk for allergic sensitization. In contrast, maternal smoking (OR: 0.469; P < 0.005), increasing parity (OR: 0.881; P < 0.05), maternal pre-pregnancy overweight (OR: 0.742; P < 0.005) and preterm birth (OR: 0.484; P < 0.05) decreased the risk for allergic sensitization.

**Conclusions:**

In addition to supporting previous findings, this study is first to report an increased risk of allergic sensitization after assisted conception. Beyond this finding’s potential implementation in preventative strategies, exploration of this association could further pathophysiological understanding of allergic disease.

## Introduction

Affecting around 30–40% of the world’s population, allergies are the most common and earliest-onset non-communicable diseases [1]. With a remarkable and rapid increase in prevalence especially in industrialized countries, their prevention and treatment have become a public health priority [2].

Despite increasing interest, the pathogenesis of allergic disease remains unclear. Even though the importance of a hereditary component is well established, there is considerable evidence that the effect of family history partly reflects shared environmental risk factors [3]. Epidemiological studies emphasize the importance of early environmental factors [4]. In utero adaption to the maternal environment by means of epigenetic modifications are considered to have evolved as effective survival strategies preparing the fetus for postnatal life. However, fetal adaptation comes with the risk of a mismatch between the predicted and the actual encountered environment and is assumed to contribute to the development of allergic disease [5, 6].

This study aims to investigate the effect of several maternal and perinatal factors and their association with allergic sensitization in a pediatric population.

## Materials and methods

### Study population and design

**Fig. 1:**
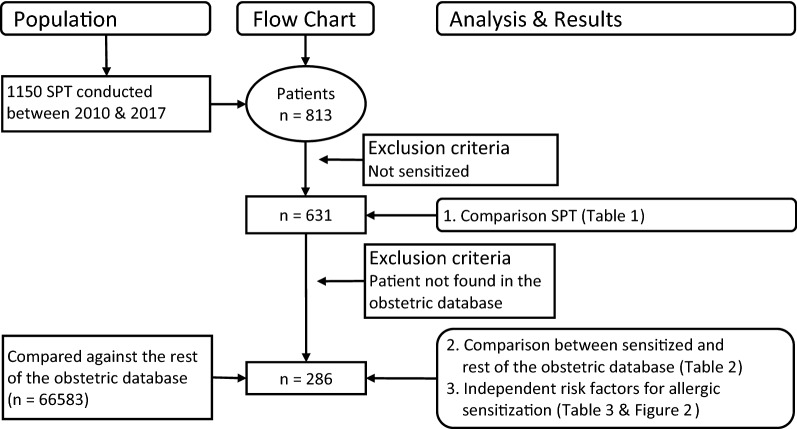
Populations used during analysis. Flow diagram summarizing the research design (population samples, sample sizes and analysis results presented in tables and figures and described in the supplemental material)

This is a retrospective study linking skin prick test (SPT) data with hospital records contained in the state-wide mandatory electronic obstetric database (Obstetrix™) [7] from a metropolitan teaching hospital in Western Sydney, Australia. SPTs conducted on children attending Nepean Hospital between January 2010 and December 2017 were reviewed. 286 atopic children with a positive SPT were linked with Obstetrix™ ranging from 2000 to 2017 (n = 67,268), using medical record numbers. After exclusion of stillbirths (n = 399) from Obstetrix™, maternal and perinatal characteristics of SPT positive children (n = 286) were compared with the remaining children who did not undergo allergy assessment (n = 66,583), as shown in Fig. 1. The rate of atopy in the remainder of the obstetric cohort was assumed to be either similar, or lower than the average rate of atopy in Australian children (approximately 20%) [8]. For more information regarding data collection see Supplementary Information.

### Skin prick testing

The allergens tested were selected following specialist consultation for suspected allergic disease. Standard commercial food and aeroallergen extracts were used as recommended by manufacturers (Additional file 1: Table S1). Skin prick testing and interpretation was carried out following Australasian Society of Clinical Immunology and Allergy (ASCIA) recommendations [9].

### Anthropometric measurements

As birth weight, length and head circumference (HC) are naturally confounded by gestational age and sex, z-scores were used for further analysis. Multiple births and stillbirths were excluded from the data, leaving 64,204 entries. Entries beyond three standard deviations were regarded entry errors and excluded from further calculation. The LMS parameters described by Cole [10] were then calculated based on 32,460 weight, 30,666 length and 31,526 HC entries for boys and 30,171 weight, 27,671 length and 29,480 HC entries for girls. No z-scores were calculated for entries < 26 weeks gestation (n = 301) and > 42 weeks gestation (n = 140).

### Statistical analysis

All statistical analyses were performed using SPSS statistics (version 24, IBM Corporation, Chicago, Illinois, USA). Descriptive statistics are reported as means and standard deviation (mean ± SD) for normally distributed data, non-normally distributed data are reported as median and interquartile ranges [median (IQR)]. Normality was assessed using histograms. Descriptive statistics of categorical variables were reported as frequency and percentage, or only percentage. Continuous outcomes were compared between groups using t-tests or Mann–Whitney U-tests. Categorical outcomes were compared using chi-square or Fisher exact tests. Tests were two-sided. To select predictive factors for allergic sensitization, binary logistic regression was carried out. Backward elimination, excluding variables with a significance level of P ≥ 0.1, ensured that the most parsimonious multivariable model was selected. To ensure that the binary logistic regression model’s assumption of variable independency was not violated, variance inflation factors (VIFs) for all variables in the final regression model were calculated. Significance was assumed at P < 0.05.

## Results

### Characteristics of atopic children

Firstly, we compared children with positive SPT and available obstetric data (included children), with children with positive SPT for which obstetric data was not available (excluded children), Table 1. Generally, children with positive SPTs were challenged for [median (IQR)] 19 [11–27] allergens. While excluded children were sensitized to a similar number of allergens, they were significantly older and more frequently sensitized to inhaled allergens than included children. Because there was no obstetric data available prior to the year 2000, this study naturally excluded older children. The sensitization pattern for inhaled allergens has in previous studies been associated with increasing age [11], which might explain these differences. The sensitization frequencies to individual allergens for included children are shown in Additional file 1: Table S2.

**Table 1:** Characteristics for positive SPT with and without available obstetric data

Variable	Obstetric and pos. SPT^a^		Pos. SPT^b^ only		P
	Median	IQR	Median	IQR	
Age at test (years)	3.47	1.70–7.34	5.56	2.51–9.49	< 0.001*
n allergens tested	19	11–27	19	11–27	0.978*
Pos. allergens	4	2–7	4	2–8	0.213*
n pos. inh. allergens	2	0–3	2	1–4	0.019*
n pos. ing. allergens	2	0–5	2	0–5	0.616*

	n	%	n	%	P
Sex: male	171	59.8	191	55.4	0.336**
n SPTs per patient					0.145**
1	193	67.5	256	74.2	
2	51	17.8	52	15.1	
3	24	8.4	18	5.2	
4+	18	6.4	19	5.5	
Children sensitized to inh.	195	68.2	265	76.8	0.013**
Children sensitized to ing.	210	73.4	241	69.9	0.309**
Cross-sensitized children	118	41.3	161	46.7	0.197**

*pos.* positive, *IQR* interquartile range, *inh.* inhaled, *ing.* ingested, cross-sensitized, sensitized to both ing. and inh. allergens

* Mann–Whitney U test; ** chi-square test

^a^(n = 286); ^b^(n = 345)

### Comparison of perinatal outcomes

Comparison of maternal and perinatal characteristics between atopic children and children born in the same time period (Table 2) showed that atopic children were more frequently male (P < 0.01). The high frequency of males in the sensitized sub-population explains why birth weight (mean ± SD; 3.395 ± 0.591 kg vs. 3.305 ± 0.685 kg; P < 0.05) was generally higher in SPT positive children, while z-scores did not differ between groups.

**Table 2:** Comparison of obstetric outcomes between sensitized children and general population

Infant related factors	Sensitized children (n = 286)			Rest of population (n = 66,583)			
	n			n			P
Sex (Male)	286	171	59.8%	66,576	34,327	51.6%	0.005*
Season of birth	286			66,583			0.806*
Spring		66	23.1%		16,241	24.4%	
Summer		66	23.1%		16,086	24.2%	
Autumn		73	25.5%		17,005	25.5%	
Winter		81	28.3%		17,251	25.9%	
Preterm (Yes)	286	19	6.6%	66,583	7701	11.6%	0.009*
Breastfed (Yes)	250	208	83.2%	49,619	38,431	77.5%	0.030*
Birth weight (kg)	286	3.395	± 0.591	66,574	3.305	± 0.685	0.011**
Birth length (cm)	279	50.5	± 3.4	64,870	50.3	± 3.9	0.279**
Birth HC (cm)	280	34.5	± 2.1	65,220	34.3	± 2.3	0.053**
z-score birth weight	286	0.062	± 1.014	65,852	0.007	± 1.072	0.387**
z-score birth length	279	0.154	± 1.109	63,933	0.134	± 1.184	0.780**
z-score birth HC	280	0.022	± 0.974	64,455	0.007	± 1.075	0.798**
Pregnancy related factors							
Conception (assisted)	242	20	8.3%	48,861	1960	4.0%	0.001*
TPL (yes)	250	9	3.6%	50,066	2403	4.8%	0.639*
Medical problem during pregnancy (yes)	252	11	4.4%	50,242	1934	3.8%	0.671*
Resuscitation intervention (yes)	251	84	33.5%	50,303	15,752	31.3%	0.463*
Delivery techniques used (yes)	251	14	5.6%	50,302	2206	4.4%	0.358*
Mode of birth (CS)	251	94	37.5%	50,302	17,188	34.2%	0.274*
Multiple birth (yes)	286	10	3.5%	66,583	2610	3.9%	0.713*
Maternal factors							
Gravidity	286	1	(0–2)	66,575	2	(1–3)	< 0.001***
Parity	286	1	(0–1)	66,579	1	(0–2)	0.001***
BMI (kg/m^2^)	243			49,106			0.017*
Underweight (< 18.5)		33	13.6%		5234	10.7%	
Normal (18.5–24.9)		120	49.4%		20,119	41.0%	
Overweight (25–29.9)		49	20.2%		11,531	23.5%	
Obesity class I (30–34.9)		24	9.9%		6676	13.6%	
Obesity class II (35–39.9)		9	3.7%		3193	6.5%	
Obesity class III (≥ 40)		8	3.3%		2353	4.8%	
Age at delivery (years)	286			66,582			0.001*
< 20		13	4.5%		4382	6.6%	
20–35		211	73.8%		52,801	79.3%	
> 35		62	21.7%		9399	14.1%	
Diabetes (Yes)	286	19	6.6%	66,583	3988	6.0%	0.642*
Smoking (Yes)	242	24	9.9%	48,920	10,348	21.2%	< 0.001*
Alcohol consumption (Yes)	242	1	0.4%	48,797	877	1.8%	0.139****
Illegal drug use (Yes)	242	3	1.2%	48,861	1133	2.3%	0.387****
Born in Australia (Yes)	286	201	70.3%	66,578	50,849	76.4%	0.015*

The following binary variables are defined as: sex, male or female; type of conception, spontaneous or assisted; mode of birth, vaginal or CS. All other binary variables were defined as yes or no. BMI categories are according to WHO. Values are displayed as either n (%), median (IQR) or mean ± SD

*HC* head circumference, *TPL* threatened premature labour, *CS* caesarean section

* Chi-square test; ** t-test; *** Mann–Whitney U test; **** Fishers exact test

Mothers of atopic children had more frequently received assisted conception (P < 0.005), were less likely to smoke (P < 0.001), had fewer prior gravidities (P < 0.001) and consequently a lower parity (P < 0.005, Table 2). Interestingly, mothers of sensitized offspring also were older at delivery (P < 0.005) and more frequently born outside of Australia (P < 0.05). Generally, assisted conception and maternal age were significantly correlated (Pearson correlation r = 0.095, P < 10^−98^, Additional file 1: Figure S1). When stratifying our data by maternal origin of birth, it became apparent that atopic offspring more frequently had a mother born in Asia (19.1% vs. 10.2%; P < 10^−6^), while other children born in the same time period more frequently had mothers born in Australia (76.7% vs. 71.3%; P < 0.05; Additional file 1: Table S3) or in the rest of Oceania (7.1% vs. 3.2%; P < 0.05). Positive SPT frequency did not differ between children to European mothers (4.3% vs 3.3%; P = 0.36), North American mothers (0.4% vs. 0.4%; P = 0.84), South American mothers (1.1% vs 0.4%; P = 0.05) and mothers from Africa (0.7% vs. 1.9%; P = 0.14).

### Independent risk factors for allergic sensitization

**Fig. 2:**
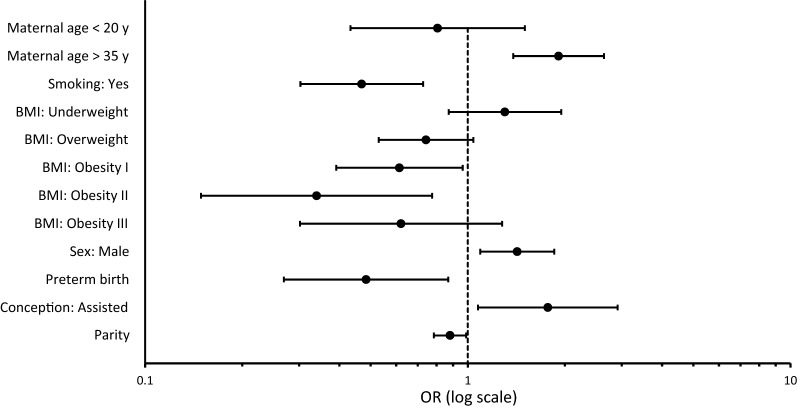
Independent risk factors for allergic sensitization. Odds ratio (95% CI) for allergic sensitization to inhaled and ingested allergens. Out of 23 predictors entered in the initial regression model the seven factors with the most significant association to allergic sensitization were selected through backward elimination. Reference categories: Maternal age, age 20 to 35; smoking during pregnancy, no smoking; maternal BMI, normal weight (18.5 to 24.9); infant sex, female; preterm birth, term birth (≥ 37 gestation); conception type, spontaneous

All variables in Table 2 were entered in the logistic regression model, except for gravidity, birth weight, length and HC. Gravidity was excluded because of the high correlation to parity; z-scores were used instead of birth measurements, to avoid confounding by sex and gestational age. After backward elimination, seven predictors remained in the model (Table 3; Fig. 2), namely maternal age, smoking maternal BMI, infant sex, preterm birth, parity, conception type and parity. The independence of these risk factors was ensured by calculation of VIFs for all independent variables in the final binary logistic regression model (Additional file 1: Table S4).

**Table 3:** Logistic regression with backward elimination

Predictive variable	OR	95% CI	P
Maternal age			< 0.001
< 20 years	0.806	0.433–1.503	
20 to 35	Reference		
> 35 years	1.912	1.383–2.642	
Maternal smoking: yes	0.469	0.303–0.727	0.001
Maternal BMI			0.004
Underweight	1.303	0.873–1.947	
Normal weight	Reference		
Overweight	0.742	0.530–1.040	
Obesity class I	0.614	0.391–0.964	
Obesity class II	0.340	0.149–0.775	
Obesity class III	0.621	0.302–1.278	
Infant sex: male	1.423	1.093–1.854	0.009
Preterm birth: yes	0.484	0.269–0.870	0.015
Conception: Assisted	1.771	1.076–2.914	0.025
Parity	0.881	0.785–0.988	0.031

R^2^ = 0.03. P < 10^−10^. Mode of conception was defined as assisted or spontaneous

*OR* odds ratio, *CI* confidence interval

To ensure that these results did not only arise from the heightened sensitivity seen when a small population is compared to a bigger one, we computed binary logistic regressions on five randomly selected subpopulations, similar in size to the group with sensitized children (comprising ca 0.4% of the Obstetrix™ cohort, Additional file 1: Table S5). This analysis showed that results most likely are not a product of increased sensitivity. We also investigated whether time-dependent trends in the populations might have confounded analysis, since the birth year distribution of atopic children in our study slightly deviated from the distribution of the remaining population (Additional file 1: Figure S2). For this purpose, we investigated collinearity between birth year and factors identified in the binary logistic regression (Table 3). Overall correlation strength between SPT outcome and factors from the binary logistic regression remained unchanged when adjusted for birth year (Additional file 1: Table S6). To further ensure that results from the binary logistic regression model were not confounded, we forced birth year together with all previously identified factors (Table 3) into the regression model. This did not increase the regression models R^2^ and OR as well as P values of all previously identified factors remained largely unchanged, indicating that birth year distribution has not affected our results (Additional file 1: Table S7).

## Discussion

Prior work has established the importance of perinatal and maternal factors in the development of allergic disease. Godfrey et al. for example reported that disproportionate fetal growth was associated with raised IgE concentrations in adult life [4]. However, these studies mostly focused on anthropometric measurements, which are potentially confounded by gestational age and infant sex. In this study, we tested the extent to which maternal factors and routinely measured perinatal factors could be associated with the development of allergic sensitization in children. Aside from strengthening the association of previously suggested risk factors of allergic sensitization, one novel perinatal factor was found to have an effect on childhood atopy, namely assisted conception.

While few previous studies have investigated the effect of assisted conception and/or maternal subfertility on allergic disease, our study is the first to report an increased risk of allergic sensitization in offspring after assisted conceptions. In accordance with our findings, some prior studies have reported an association between assisted conceptions/maternal subfertility and asthma [12–14]. Noteworthy, however, is that preceding studies used questionnaires, diagnosis codes, or prescription of antihistamines to identify atopic patients. While antihistamines can be used for treatment of other diseases, and diagnosis codes and questionnaires don’t guarantee underlying allergic status, the use of SPTs is a major strength of this study.

Assisted conception was found to be mildly correlated to maternal age. Though slightly correlated both variables remained in the binary logistic regression model as predictors after backward elimination, thus indicating that they have independent effects on allergic sensitization status during childhood. We further established that the assumptions of the binary logistic regression were not violated by the co-existence of these two variables in the model (Additional file 1: Table S4). Furthermore, we show that this finding is not due to time-dependent changes of the availability of assisted conception (Additional file 1: Figure S2, Tables S6, S7). Therefore, we assume that our analysis identified a true effect of assisted conception on pediatric allergic sensitization status. Though this has to be evaluated carefully in future studies, we hypothesize that such an effect might be explained by immunological shifts in the placenta during pregnancy.

Notable is that this study fails to identify differences in anthropometric measurements between sensitized children and the general population, after adjusting for gestational age and sex. This is not surprising, since a comprehensive review [15] pointed out, that methodological differences between previous studies have made the relationship between anthropometric measurements and allergic sensitization ambiguous. However, our study shows that both gestational age and infant sex, were significantly associated with allergic sensitization. While the association of male sex with increasing risk for allergic disease during early childhood is established [16], our study provides additional support to the fairly new concept that prematurity may decrease the risk of allergic sensitization in childhood.

Even though findings are heterogeneous, evidence that prematurity might be associated with a lower prevalence of allergic sensitization is accumulating. Two large Swedish register studies [17, 18] report that prematurity is associated with a lower prevalence of allergic rhinitis, in agreement with our findings. The relationship between gestational age and prevalence of allergic sensitization might be explained by hormone dependent shift in the placental immune environment. It can be hypothesized that a lower gestational age is associated with lower explosibility for progesterone which in turn leads to a less Th2 predominant environment in utero [19]. Alternatively, it might reflect the effect of underlying risk factors of premature delivery.

In parallel with some previous research, we find that advanced maternal age (> 35 years) is a risk factor for allergic sensitization in childhood. As proposed by Dowhower Karpa et al. [20], increasing maternal age might expose offspring to a greater risk for atopic disease due to age-dependent changes in the gut’s microbiome, in particular the decrease of Lactobacilli and the increase in Clostridia have been linked to increased risk for atopy in the offspring.

Our results also strengthen the widely established association of increasing parity/birth order as a protective factor. Previous studies have suggested that increasing parity leads to in utero programming, which results in a more anti-inflammatory intrauterine environment, reflected by lower cord blood IgE levels at birth [21].

In our study, we found that mothers of sensitized children were less frequently overweight and smoked less. Therefore, our regression model suggests that maternal pre-pregnancy overweight and maternal smoking might be associated with a lower prevalence of allergic disease (Fig. 2). In regards to maternal BMI, some preceding studies suggested that the relationship of maternal BMI to allergic disease was U-shaped [22], implying that maternal underweight as well as maternal overweight were associated with a higher risk for atopy. Since most previous studies identifying maternal overweight as a risk factor, were conducted in the northern hemisphere, it is possible that differences in diet, or gut microbiome, could explain that maternal overweight was associated with a lower prevalence of allergic sensitization in this study. Furthermore, some previous studies [23] suggested that maternal smoking might be associated with a lower prevalence of allergic sensitization. Though the majority of scientific reports indicate that smoking and obesity are associated with a increased prevalence of allergic sensitization in offspring [24, 25]. A possible explanation for this studies deviating results might therefore be that, since a family history of allergic disease is a strong predictive factor for allergic sensitization [26], mothers of sensitized children are likely to opt for a healthier lifestyle motivated by their own allergic disease.

Interestingly, positive SPT were more common in offspring of mothers born in Asia. This supports previous findings made by the HealthNuts study group [27] and could suggest that maternal continent of birth affects the development of atopy.

We compared children with confirmed allergic sensitization with a population including approximately 20% sensitized children [8]. While this aggravated identification of risk factors, it also ensured that the risk factors identified differed strongly between groups and hence should be repeatable in other populations. Furthermore, the use of z-scores is a strength of this study, as they were calculated based on a geographically and contemporaneously matched population. Another major strength of this study is the use of SPT to quantify allergic sensitization. However, the retrospective design did not allow controlling for allergic family history, maternal education or socioeconomic status, which in previous reports have been associated with allergic disease [16, 26]. Furthermore, this was a monocentric study and while the population size in this study is comparable to many other studies in this field, larger multicenter cohorts are needed to confirm these findings. It should also be mentioned again that children in the presented population underwent SPT due to clinical indications and hence were not screened with standard allergen panels. While clinical indication for SPT allows us to identify a group of children with symptomatic allergic sensitization, the absence of standard panels complicates comparison between groups. Furthermore, some previous studies [11], as well as the comparison between included and excluded children presented here (Table 1), indicate that sensitization pattern of children might be age dependent, though this dimension was not considered in the present study it should be considered in future studies of pediatric populations.

This study has identified associations between perinatal and maternal factors and allergic sensitization. In part our results confirm some previous reports but add to the current literature by using an objective measure of allergic sensitization and applying a multiple regression backward elimination approach. Interestingly, we show an increased risk of atopy after assisted conception and in offspring of mothers born in Asia, neither of which have been described in a more general context of allergic sensitization. These findings support the notion that early periconceptional and other environmental factors are likely to predispose towards atopy and warrant further mechanistic and epidemiological studies, aiming to develop primary preventive strategies for childhood allergies.

## Supplementary information

**Additional file 1.** Additional methods. **Table S1.** Compilation of allergens used in skin prick tests. **Table S2.** Skin prick test based sensitization rates for the 286 included children. Only allergens for which at least one child tested positive are shown. **Table S3.** Continent of birth for mothers of atopic children and children from the remaining population. **Table S4.** Correlation coefficients between independent risk factors for allergic sensitization. **Table S5.** Binary logistic regression of independent risk factors from Table 3 comparing a randomly chosen subpopulation consisting of 0.4% children to the remaining cohort. **Table S6.** Correlation of birth year with the outcomes of the binary logistic regression model. **Table S7.** Modelling the effect of birth year on the outcomes of the binary logistic regression model (Table 3). **Figure S1.** Histogram of maternal age in the context of conception type. **Figure S2.** Histogram of birth year for sensitized children and the remaining population.

## Data Availability

The data analysed in this study was obtained from hospital records. The datasets for this manuscript are not publicly available to preserve patient integrity and confidentiality. Requests to access the datasets should be directed to the Nepean Blue Mountains Local Health District.

## Abbreviations

BMI: Body mass index
HC: Head circumference
IgE: Immunoglobulin E
IQR: Interquartile range
OR: Odds ratio
SD: Standard deviation
SPT: Skin prick test
VIF: Variance inflation factor
