# Supplementary material for: Analysis of maternal and perinatal determinants of allergic sensitization in childhood
Source: Allergy Asthma Clin Immunol. 2020 Jul 31;16:71. doi: 10.1186/s13223-020-00467-5 (PMC7477859; doi:10.1186/s13223-020-00467-5)

Analysis of Maternal and Perinatal Determinants of Allergic Sensitization in Childhood

Samuel Schäfer^1^, Anthony Liu^2 *^, Dianne Campbell^3 4^, Ralph Nanan^2^

^1^ Department of Clinical and Experimental Medicine, Linköping University, Linköping, Sweden

^2^ Discipline of Paediatrics and Child Health, Charles Perkins Centre - Nepean, Sydney Medical School - Nepean, The University of Sydney, Penrith, NSW, Australia

^3^ Immunology and Allergy, The Children's Hospital at Westmead, Westmead, NSW, Australia.

^4^ Discipline of Child and Adolescent Health, The University of Sydney, Sydney, NSW, Australia

* Correspondence: anthony.liu@health.nsw.gov.au

Additional Information

# Additional Methods

# Skin Prick Tests

Patients were referred for possible allergic conditions to the hospital’s specialist allergy outpatient clinic. 1150 SPTs were conducted between 2010 and 2017 (**Fig 1**). For patients with repeated testing’s (n=194), SPTs were analyzed individually and then outcomes were pooled, to ensure that every patient only entered the analysis once (n=813). During pooling process all allergens that the patient had been tested for were compiled, then sensitization was inferred if the patient ever had a positive skin prick reaction to this allergen. Repeated tests were usually performed to widen the allergen spectrum of most patients (94.8%, n=184) and normally occurred within three months after the initial test.

**Obstetrix^TM^**

Obstetrix^TM^ contained records of 67268 (51.6% males) deliveries at the Nepean Hospital between 2000 and 2017. From records in Obstetrix^TM^ information on maternal health (gravidity - number prior gravidities, parity - number of prior pregnancies exceeding 24 weeks gestation, etc.), pregnancy related information (type of conception, mode of delivery, etc.) and infant-centered outcomes (sex, gestational age, etc.) could be extracted. Preterm birth was defined as birth before 37 weeks gestation. Mode of feeding was categorized into breastfed (even when combined with infant formula) and formula fed and based on the medical records feeding entry at discharge. Type of conception was categorized into spontaneous and assisted; where assisted conception is defined as conception during any kind of infertility treatment. Stillbirths (n=399, 51.4% males) were excluded from further analysis.

Obstetric data was successfully obtained for 286 children with positive SPTs, the remaining children with positive SPTs were not delivered at the Nepean hospital and/or had been born prior to the year 2000 and were therefore excluded. Since no standard panels were used, children with negative SPT were not used as a control group, but rather the atopic children were compared to the remainder of the obstetric database (**Fig 1**) to ensure the generalization potential of our findings.

# Additional Tables

**Table S1. Compilation of allergens used in skin prick tests**

| **Standard allergens** | | | | | | | | | | |
| --- | --- | --- | --- | --- | --- | --- | --- | --- | --- | --- |
| **Allergen** | **Concentration** | **Manufacturer** | |  | **Allergen** | | **Concentration** | | **Manufacturer** | |
| Diluent |  | ALK | |  | Baker’s yeast | | Actual yeast used | |  | |
| Histamine | 10mg/mL | ALK | |  | Tuna | | 1:10 W/V | | ALK | |
| Rye grass | 50 HEP/mL | Immunotek | |  | Salmon | | 2000 µg/mL | | Immunotek | |
| Plantain (English) | 50 HEP/mL | Immunotek | |  | Codfish | | 2000 µg/mL | | Immunotek | |
| Cat hair | 50 HEP/mL | Immunotek | |  | Shrimp | | 1:20 W/V | | ALK | |
| Dog hair | 10 µg/mL | Immunotek | |  | Crab mix | | 1:20 W/V | | ALK | |
| HDM (Pteronys) | 30 HEP/mL | ALK | |  | Sardine | | 1000 IC/mL | | Alyostal | |
| HDM (Farinae) | 30 HEP/mL | ALK | |  | Mussel | | 2000 µg/mL | | Immunotek | |
| Alternaria | 1:10 W/V | ALK | |  | Oyster | | 1:10 W/V | | ALK | |
| Aspergillus fumigatus | 1:10 W/V | ALK | |  | Hazelnut | | 750 µg/mL | | Immunotek | |
|  |  |  |  |  | Almond | | 1:20 W/V | | ALK | |
| Aspergillus niger | 1:10 W/V | ALK | |  | Walnut | | 1:20 W/V | | ALK | |
| Cockroach mix | 1:100 W/V | ALK | |  | Cashew | | Actual cashew | |  | |
| Latex | 500 µg/mL | ALK | |  | Pecan | | 1:10 W/V | | ALK | |
| Horse hair | 50 µg/mL | Immunotek | |  | Pistachio | | 1:10 W/V | | ALK | |
| Birch mix | 30 HEP/mL | ALK | |  | Macademia | | Actual macademia | | | |
| Ragweed | 1:100 W/V | ALK | |  | Pine nut | | 750 µg/mL | | Immunotek | |
| Grass mix | 100,000 BAU/mL | ALK | |  | Brazil nut | | 1:10 W/V | | ALK | |
| Cow milk | Actual cow milk |  | |  | Barley | | 1:10 W/V | | ALK | |
| Wheat flour | 1:20 W/V | ALK | |  | Corn | | 1:10 W/V | | ALK | |
| Peanut mix | 1:10 W/V | ALK | |  | Rice | | 1:10 W/V | | ALK | |
| Egg yolk | 1:100 W/V | ALK | |  | Rye grain | | 750 µg/mL | | Immunotek | |
| Egg white | 1:100 W/V | ALK | |  | Kiwi | | 1:10 W/V | | Immunotek | |
| Soybean | Actual soybean | | |  | Tomato | | 1:10 W/V | | ALK | |
| Sesame seed | 1:10 W/V | ALK | |  | Strawberry | | 1:10 W/V | | ALK | |
| Wheat grain | 1:10 W/V | ALK | |  |  | |  | |  | |
| **Other allergens requested by physician** | | | | | | | | | | |
| Apple | Apricot | | Avocado | | | Banana | | Barramundi | | Basa fillet |
| Beef | Black berry | | Blueberry | | | Bream | | Brie cheese | | Broccoli |
| Brown onion | Calamari | | Carrot | | | Cherry | | Chickpeas | | Chili |
| Coconut | Coriander | | Eggplant | | | Garlic | | Goat meet | | Grape |
| Greek yoghurt | Green capsicum | | Guinea pig | | | Honey | | Lamb meet | | Lemon |
| Lupin | Lychee | | Mandarin | | | Mango | | Nectarine | | Nutella |
| Octopus | Orange | | Ovaltine | | | Paprika | | Pear | | Penicillin |
| Pepper | Peppermint | | Pineapple | | | Plum | | Poppy seeds | | Pork |
| Potato | Pumpkin | | Raspberry | | | Red capsicum | | Rockmelon | | Shallots |
| Squid | Sunflower | | Sultana | | | Sweet & sour sauce | | Tahini | | Tamarillo |
| Vanilla | Vegemite | | Water melon | | | Weetabix | | White nectarine | | Whiting |
| Yellow-capsicum | Yellow split pea | | Zucchini | | |  | |  | |  |

**Table S2. Skin prick test based sensitization rates for the 286 included children. Only allergens for which at least one child tested positive are shown.**

| **n children** | |  |  |  | **n children** | |  |  |
| --- | --- | --- | --- | --- | --- | --- | --- | --- |
| **Positive** | **Tested** | **Sensitization frequency (%)** | **Allergen** |  | **Positive** | **Tested** | **Sensitization frequency (%)** | **Allergen** |
| 139 | 236 | 58,90 | HDM (pterony) |  | 12 | 79 | 15,19 | Grass mix |
| 133 | 235 | 56,60 | HDM (farina) |  | 10 | 104 | 9,62 | Alterinae |
| 99 | 209 | 47,37 | Peanut |  | 9 | 56 | 16,07 | Crab |
| 96 | 192 | 50,00 | Egg white |  | 7 | 40 | 17,50 | Codfish |
| 85 | 195 | 43,59 | Egg yolk |  | 5 | 46 | 10,87 | Latex |
| 63 | 219 | 28,77 | Rye grass |  | 5 | 13 | 38,46 | Kiwi |
| 60 | 179 | 33,52 | Cashew |  | 4 | 16 | 25,00 | Salmon |
| 58 | 177 | 32,77 | Pistachio |  | 4 | 9 | 44,44 | Corn |
| 53 | 218 | 24,31 | Cat |  | 3 | 94 | 3,19 | Yeast |
| 48 | 165 | 29,09 | Hazelnut |  | 2 | 9 | 22,22 | Tomato |
| 47 | 169 | 27,81 | Walnut |  | 2 | 8 | 25,00 | Rye |
| 46 | 177 | 25,99 | Almond |  | 2 | 3 | 66,67 | Watermelon |
| 45 | 221 | 20,36 | Plantain |  | 2 | 2 | 100,00 | Pea |
| 40 | 178 | 22,47 | Pine nut |  | 2 | 2 | 100,00 | Vegemite |
| 39 | 203 | 19,21 | Dog |  | 1 | 6 | 16,67 | Lupin |
| 38 | 179 | 21,23 | Pecan |  | 1 | 5 | 20,00 | Rice |
| 36 | 110 | 32,73 | Macademia |  | 1 | 5 | 20,00 | Banana |
| 34 | 178 | 19,10 | Brazil nut |  | 1 | 3 | 33,33 | Coconut |
| 31 | 154 | 20,13 | Cockroach |  | 1 | 2 | 50,00 | Mango |
| 26 | 150 | 17,33 | Milk |  | 1 | 1 | 100,00 | Potato |
| 21 | 177 | 11,86 | Soybean |  | 1 | 1 | 100,00 | Rockmelon |
| 20 | 95 | 21,05 | Birch |  | 1 | 1 | 100,00 | Pumpkin |
| 19 | 148 | 12,84 | Wheat |  | 1 | 1 | 100,00 | Nectarin |
| 18 | 156 | 11,54 | Sesame |  | 1 | 1 | 100,00 | Raspberry |
| 16 | 92 | 17,39 | Ragweed |  | 1 | 1 | 100,00 | Fish sauce |
| 15 | 142 | 10,56 | Horse |  | 1 | 1 | 100,00 | Yellow split pea |
| 15 | 118 | 12,71 | Tuna |  | 1 | 1 | 100,00 | Apple |
| 15 | 58 | 25,86 | Oyster |  | 1 | 1 | 100,00 | Sultana |
| 14 | 57 | 24,56 | Mussel |  | 1 | 1 | 100,00 | Natural yoghurt |
| 13 | 66 | 19,70 | Wheat grain |  | 1 | 1 | 100,00 | Cream |
| 13 | 53 | 24,53 | Shrimp |  | 1 | 1 | 100,00 | Strawberry yoghurt |
| 13 | 53 | 24,53 | Sardine |  | 1 | 1 | 100,00 | Vanilla yoghurt |

**Table S3. Continent of birth for mothers of atopic children and children from the remaining population. Data was available for 282 (98.6%) atopic children and 66283 (99.5%) children from the remaining population. First, a Chi-Square test was conducted, indicating a difference between groups (P < 10^-5^). To stratify this outcome, additional two-level Chi-Square tests were calculated for every individual maternal origin.**

Abbreviations: SPT; skin prick test.

| **Mother born in** | **SPT+ group**  **n (%)** | **Control group**  **n (%)** | **P*** |
| --- | --- | --- | --- |
| Australia | 201 (71.3%) | 50848 (76.7%) | 0.031 |
| Asia | 54 (19.1%) | 6773 (10.2%) | < 10^-6^ |
| Europe | 12 (4.3%) | 2177 (3.3%) | 0.362 |
| North America | 1 (0.4%) | 290 (0.4%) | 0.833 |
| South America | 3 (1.1%) | 240 (0.4%) | 0.051 |
| Africa | 2 (0.7%) | 1267 (1.9%) | 0.141 |
| Rest of Oceania | 9 (3.2%) | 4688 (7.1%) | 0.011 |
| Valid cases | 282 (100%) | 66283 (100%) |  |
| Missing cases | 4 | 300 |  |
| Total cases | 286 | 66583 |  |

**Table S4. Correlation coefficients between independent risk factors for allergic sensitization. VIF calculated based on a multiple regression model corresponding to the binary logistic model used in Table 3.**

Abbreviations: VIF, variance inflation factor.

Variable coding: BMI; *1 = Underweight (BMI < 18.5), 2 = Normal weight (BMI 18.5 – 24.9), 3 = Overweight (BMI 25.0 – 29.9), 4 = Class I obesity (BMI 30.0 – 34.9), 5 = Class II obesity (BMI 35.0 – 39.9), 6 = Class III obesity (BMI ≥ 40)*. Sex; *0 = Female, 1 = Male*. Conception type; *0 = Spontaneous, 1 = Assisted conception*. Smoking; *0 = No, 1 = Yes*. Preterm birth; *0 = No, 1 = Yes*. Maternal age; *1 = under 20 years, 2 = 20 to 35 years, 3 = over 35 years*. Parity; *continuous variable*.

|  | **BMI** | **Sex** | **Conception type** | **Smoking** | **Preterm birth** | **Maternal age** | **Parity** | **VIF** |
| --- | --- | --- | --- | --- | --- | --- | --- | --- |
| **BMI** | 1.000 | -0.011 | -0.030 | 0.037 | 0.038 | -0.059 | -0.086 | 1.019 |
| **Sex** | -0.011 | 1.000 | 0.001 | 0.001 | -0.002 | 0.001 | 0.011 | 1.000 |
| **Conception type** | -0.030 | 0.001 | 1.000 | 0.045 | -0.087 | -0.117 | 0.116 | 1.035 |
| **Smoking** | 0.037 | 0.001 | 0.045 | 1.000 | -0.043 | 0.123 | -0.175 | 1.045 |
| **Preterm birth** | 0.038 | -0.002 | -0.087 | -0.043 | 1.000 | -0.029 | -0.004 | 1.012 |
| **Maternal age** | -0.059 | 0.001 | -0.117 | 0.123 | -0.029 | 1.000 | -0.326 | 1.146 |
| **Parity** | -0.086 | 0.011 | 0.116 | -0.175 | -0.004 | -0.326 | 1.000 | 1.165 |

**Table S5. Binary logistic regression of independent risk factors from Table 3 comparing a randomly chosen subpopulation consisting of 0.4% children to the remaining cohort. Randomization was achieved through the “compute variable interface” in SPSS.**

Abbreviations: OR, odds ratio; CI, confidence interval.

|  | | **Random sample 1** | |  | **Random sample 2** | |  | **Random sample 3** | |  | **Random sample 4** | |  | **Random sample 5** | |  | **Mean** | | |
| --- | --- | --- | --- | --- | --- | --- | --- | --- | --- | --- | --- | --- | --- | --- | --- | --- | --- | --- | --- |
| **Variable** | | OR [CI] | P |  | OR [CI] | P |  | OR [CI] | P |  | OR [CI] | P |  | OR [CI] | P |  | OR | P |  |
| **Maternal age** | | - | 0.898 |  | - | 0.160 |  | - | 0.712 |  | - | 0.921 |  | - | 0.045 |  | - | 0.547 |  |
|  | **< 20 years** | 1.09 [0.60-1.99] | 0.784 |  | 1.60 [0.99-2.61] | 0.058 |  | 0.78 [0.40-1.49] | 0.446 |  | 1.10 [0.63-1.93] | 0.740 |  | 1.70 [1.02-2.84] | 0.042 |  | 1.25 | 0.414 |  |
|  | **20 to 35 years** | - | - |  | - | - |  | - | - |  | - | - |  | - | - |  | - | - |  |
|  | **> 35 years** | 0.93 [0.62-1.40] | 0.716 |  | 0.97 [0.62-1.50] | 0.883 |  | 1.06 [0.72-1.57] | 0.777 |  | 1.05 [0.70-1.58] | 0.802 |  | 1.38 [0.93-2.06] | 0.114 |  | 1.08 | 0.658 |  |
| **Maternal smoking: Yes** | | 1.01 [0.71-1.44] | 0.953 |  | 0.88 [0.61-1.27] | 0.498 |  | 0.93 [0.66-1.32] | 0.697 |  | 0.98 [0.69-1.39] | 0.918 |  | 1.22 [0.86-1.73] | 0.257 |  | 1.00 | 0.665 |  |
| **Maternal BMI** | | - | 0.318 |  | - | 0.321 |  | - | 0.052 |  | - | 0.548 |  | - | 0.571 |  | - | 0.362 |  |
|  | **Underweight** | 0.72 [0.42-1.24] | 0.236 |  | 0.81 [0.49-1.36] | 0.431 |  | 0.95 [0.60-1.49] | 0.814 |  | 0.77 [0.47-1.26] | 0.296 |  | 0.83 [0.48-1.43] | 0.502 |  | 0.82 | 0.456 |  |
|  | **Normal weight** | - | - |  | - | - |  | - | - |  | - | - |  | - | - |  | - | - |  |
|  | **Overweight** | 1.12 [0.79-1.58] | 0.527 |  | 0.89 [0.62-1.29] | 0.550 |  | 0.57 [0.38-0.85] | 0.006 |  | 0.74 [0.52-1.07] | 0.113 |  | 1.20 [0.83-1.73] | 0.324 |  | 0.90 | 0.304 |  |
|  | **Class I obesity** | 1.15 [0.77-1.74] | 0.495 |  | 1.20 [0.80-1.81] | 0.382 |  | 0.86 [0.56-1.31] | 0.479 |  | 0.75 [0.48-1.18] | 0.219 |  | 1.02 [0.64-1.63] | 0.928 |  | 1.00 | 0.501 |  |
|  | **Class II obesity** | 0.67 [0.34-1.35] | 0.264 |  | 0.48 [0.21-1.10] | 0.083 |  | 0.64 [0.33-1.23] | 0.177 |  | 0.73 [0.39-1.36] | 0.316 |  | 0.81 [0.40-1.62] | 0.550 |  | 0.67 | 0.278 |  |
|  | **Class III obesity** | 0.61 [0.26-1.39] | 0.237 |  | 1.21 [0.64-2.28] | 0.555 |  | 1.29 [0.74-2.23] | 0.371 |  | 0.80 [0.40-1.60] | 0.529 |  | 1.46 [0.79-2.71] | 0.226 |  | 1.07 | 0.384 |  |
| **Infant sex: Male** | | 0.83 [0.63-1.10] | 0.195 |  | 0.96 [0.73-1.28] | 0.789 |  | 1.12 [0.85-1.47] | 0.431 |  | 1.16 [0.88-1.53] | 0.302 |  | 0.95 [0.71-1.27] | 0.710 |  | 1.00 | 0.485 |  |
| **Preterm birth: Yes** | | 1.43 [0.98-2.10] | 0.067 |  | 1.35 [0.91-2.02] | 0.137 |  | 1.38 [0.94-2.02] | 0.102 |  | 1.05 [0.68-1.61] | 0.829 |  | 0.98 [0.62-1.55] | 0.924 |  | 1.24 | 0.412 |  |
| **Conception: Assisted** | | 1.66 [0.93-2.96] | 0.087 |  | 0.69 [0.30-1.58] | 0.377 |  | 0.67 [0.29-1.52] | 0.338 |  | 0.48 [0.18-1.31] | 0.150 |  | 0.66 [0.27-1.62] | 0.361 |  | 0.83 | 0.263 |  |
| **Parity** | | 1.04 [0.94-1.16] | 0.414 |  | 0.89 [0.78-1.01] | 0.062 |  | 1.01 [0.91-1.12] | 0.812 |  | 0.99 [0.89-1.10] | 0.816 |  | 0.96 [0.85-1.07] | 0.447 |  | 0.98 | 0.510 |  |
| **Model** | | R^2^ = 0.006 P = 0.296 | |  | R^2^ = 0.008 P = 0.079 | |  | R^2^ = 0.006 P = 0.167 | |  | R^2^ = 0.003 P = 0.769 | |  | R^2^ = 0.005 P = 0.442 | |  |  |  | |

**Table S6. Correlation of birth year with the outcomes of the binary logistic regression model. The effect of birth year differences on the outcomes of the binary logistic model (Table 3) were investigated using (adjusted) Pearson correlations.**

Variable coding: BMI; *1 = Underweight (BMI < 18.5), 2 = Normal weight (BMI 18.5 – 24.9), 3 = Overweight (BMI 25.0 – 29.9), 4 = Class I obesity (BMI 30.0 – 34.9), 5 = Class II obesity (BMI 35.0 – 39.9), 6 = Class III obesity (BMI ≥ 40)*. Sex; *0 = Female, 1 = Male*. Conception type; *0 = Spontaneous, 1 = Assisted conception*. Smoking; *0 = No, 1 = Yes*. Preterm birth; *0 = No, 1 = Yes*. Maternal age; *1 = under 20 years, 2 = 20 to 35 years, 3 = over 35 years*. SPT outcome; *0 = no positive SPT, 1 = positive SPT*. Parity; *continuous variable*. Birth year; *continuous variable*.

|  | **Correlation with birth year** | |  | **Correlation with SPT outcome** | |  | **Correlation with SPT outcome, adjusted for birth year** | | |
| --- | --- | --- | --- | --- | --- | --- | --- | --- | --- |
|  | **r** | **P** |  | **r** | **P** |  | **r** | **P** |  |
| BMI | 0.091 | <10^-84^ |  | -0.017 | <10^-3^ |  | -0.016 | 0.001 |  |
| Sex | -0.004 | 0.399 |  | 0.012 | 0.008 |  | 0.012 | 0.008 |  |
| Conception type | 0.026 | <10^-7^ |  | 0.014 | 0.002 |  | 0.015 | 0.002 |  |
| Smoking | -0.081 | <10^-67^ |  | -0.018 | <10^-3^ |  | -0.019 | <10^-4^ |  |
| Preterm birth | 0.030 | <10^-10^ |  | -0.009 | 0.057 |  | -0.008 | 0.069 |  |
| Maternal age | 0.017 | <10^-3^ |  | 0.014 | 0.002 |  | 0.014 | 0.002 |  |
| Parity | 0.007 | 0.128 |  | -0.014 | 0.003 |  | -0.014 | 0.003 |  |

**Table S7. Modelling the effect of birth year on the outcomes of the binary logistic regression model (Table 3). For comparison the odds ratios and confidence intervals of the original backward eliminated binary logistic regression model are also shown.**

Abbreviations: OR, odds ratio; CI, confidence interval.

Variable coding: Mode of conception was defined as assisted or spontaneous. Birth year was treated as continuous variable.

|  | | **Original binary logistic regression model (Table 3)** | | |  | **Binary logistic regression model including birth year** | | |
| --- | --- | --- | --- | --- | --- | --- | --- | --- |
| **Predictive variable** | | **OR** | **95% CI** | **P** |  | **OR** | **95% CI** | **P** |
| Maternal age | |  |  | <0.001 |  |  |  | <0.001 |
|  | < 20 years | 0.806 | 0.433 - 1.503 |  |  | 0.752 | 0.404 - 1.399 |  |
|  | 20 to 35 | Reference | |  |  | Reference | |  |
|  | > 35 years | 1.912 | 1.383 - 2.642 |  |  | 1.933 | 1.410 - 2.650 |  |
| Maternal smoking: Yes | | 0.469 | 0.303 - 0.727 | 0.001 |  | 0.453 | 0.295 - 0.696 | <0.001 |
| Maternal BMI | |  |  | 0.004 |  |  |  | 0.033 |
|  | Underweight | 1.303 | 0.873 - 1.947 |  |  | 1.097 | 0.732 - 1.642 |  |
|  | Normal weight | Reference | |  |  | Reference | |  |
|  | Overweight | 0.742 | 0.530 - 1.040 |  |  | 0.716 | 0.512 - 1.000 |  |
|  | Obesity class I | 0.614 | 0.391 - 0.964 |  |  | 0.618 | 0.398 - 0.961 |  |
|  | Obesity class II | 0.340 | 0.149 - 0.775 |  |  | 0.493 | 0.250 - 0.975 |  |
|  | Obesity class III | 0.621 | 0.302 - 1.278 |  |  | 0.599 | 0.291 - 1.230 |  |
| Infant sex: Male | | 1.423 | 1.093 - 1.854 | 0.009 |  | 1.407 | 1.087 - 1.821 | 0.010 |
| Preterm birth: Yes | | 0.484 | 0.269 - 0.870 | 0.015 |  | 0.537 | 0.326 - 0.886 | 0.015 |
| Conception: Assisted | | 1.771 | 1.076 - 2.914 | 0.025 |  | 1.823 | 1.134 - 2.931 | 0.013 |
| Parity | | 0.881 | 0.785 - 0.988 | 0.031 |  | 0.868 | 0.775 - 0.971 | 0.014 |
| Birth year | | - | - | - |  | 0.956 | 0.922 - 0.990 | 0.013 |
| Model information | | R^2^ = 0.03; P < 10^-10^ | | |  | R^2^ = 0.03; P = 0.013 | | |

# Additional Figures

**Figure S1. Histogram of maternal age in the context of conception type.**

Figure text: Age distribution of 47122 mothers with spontaneous conceptions and 1980 mothers with assisted conceptions. Maternal age was found to be significant correlated to conception type (Pearson correlation r = 0.095, P < 10^-98^). In this correlation analysis, spontaneous conception was coded as 0 and assisted conception was coded as 1, maternal age was treated as a continuous variable.


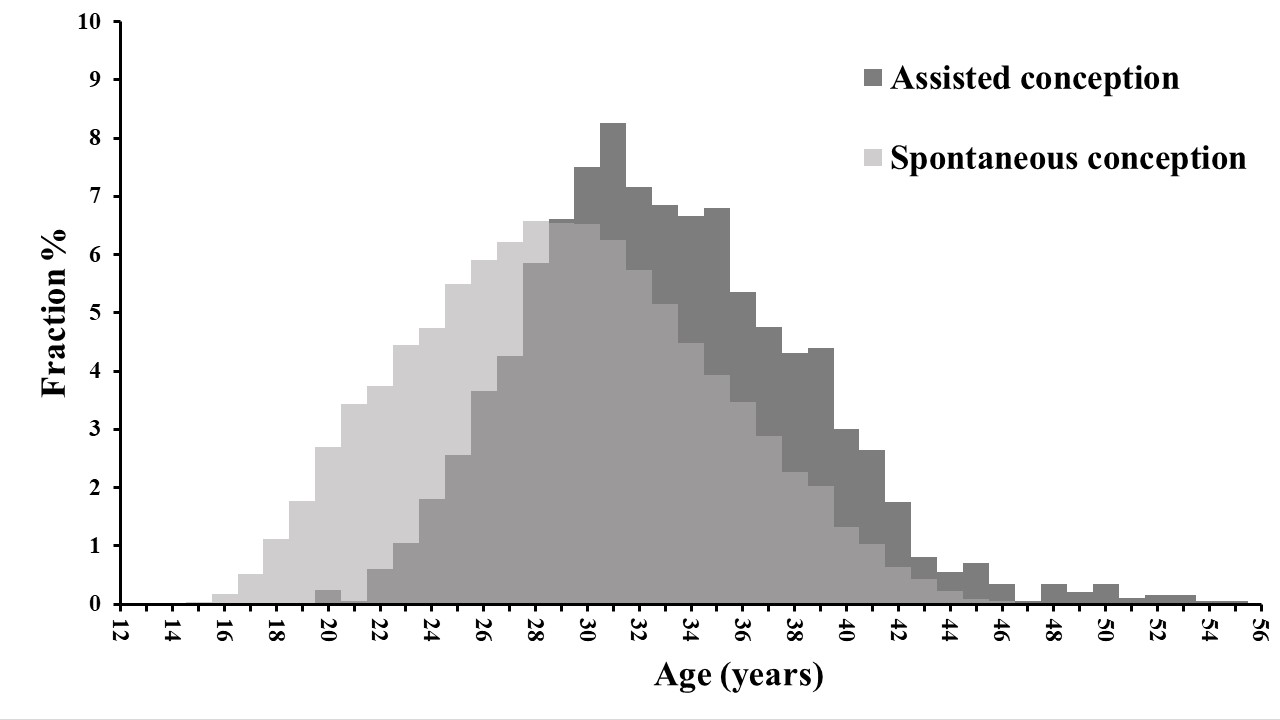


**Figure S2. Histogram of birth year for sensitized children and the remaining population.**

Figure text: Birth year distribution for included, sensitized children (n = 286) and the remaining population (n = 66583) shown in light grey and dark grey, respectively.


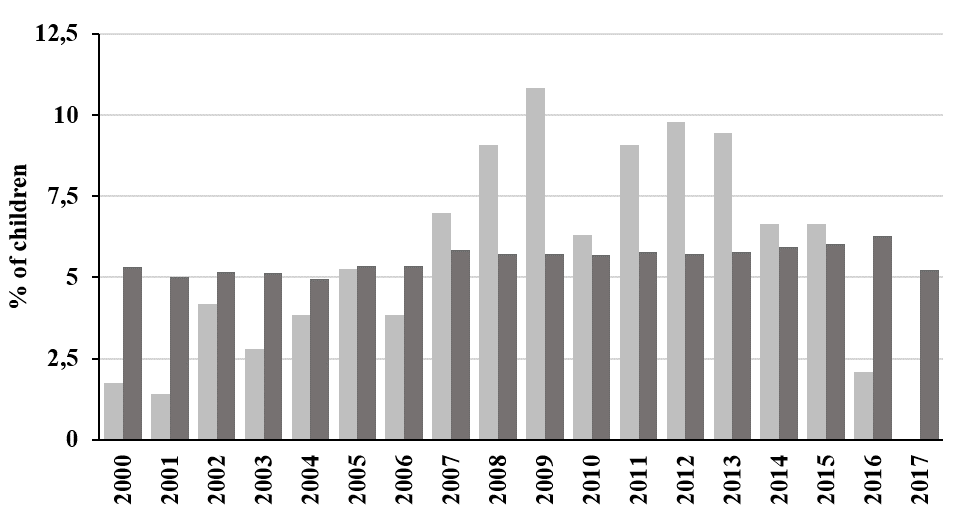

Supplement: Supplementary file 1 — Additional file 1. Additional methods. Table S1. Compilation of allergens used in skin prick tests. Table S2. Skin prick test based sensitization rates for the 286 included children. Only allergens for which at least one child tested positive are shown. Table S3. Continent of birth for mothers of atopic children and children from the remaining population. Table S4. Correlation coefficients between independent risk factors for allergic sensitization. Table S5. Binary logistic regression of independent risk factors from Table 3 comparing a randomly chosen subpopulation consisting of 0.4% children to the remaining cohort. Table S6. Correlation of birth year with the outcomes of the binary logistic regression model. Table S7. Modelling the effect of birth year on the outcomes of the binary logistic regression model (Table 3). Figure S1. Histogram of maternal age in the context of conception type. Figure S2. Histogram of birth year for sensitized children and the remaining population. [file 13223_2020_467_MOESM1_ESM.docx]
